# Protein Interaction Networks of Catalytically Active and Catalytically Inactive PqsE in Pseudomonas aeruginosa

**DOI:** 10.1128/mbio.01559-22

**Published:** 2022-09-08

**Authors:** Isabelle R. Taylor, Laura A. Murray-Nerger, Todd M. Greco, Dawei Liu, Ileana M. Cristea, Bonnie L. Bassler

**Affiliations:** a Department of Molecular Biology, Princeton University, Princeton, New Jersey, USA; b Howard Hughes Medical Institute, Chevy Chase, Maryland, USA; University Dundee; California Institute of Technology

**Keywords:** *Pseudomonas aeruginosa*, quorum sensing, virulence, protein-protein interactions, biosynthetic pathways

## Abstract

Pseudomonas aeruginosa is a human pathogen that relies on quorum sensing to establish infections. The PqsE quorum-sensing protein is required for P. aeruginosa virulence factor production and infection. PqsE has a reported enzymatic function in the biosynthesis of the quorum-sensing autoinducer called PQS. However, this activity is redundant because, in the absence of PqsE, this role is fulfilled by alternative thioesterases. Rather, PqsE drives P. aeruginosa pathogenic traits via a protein-protein interaction with the quorum-sensing receptor/transcription factor RhlR, an interaction that enhances the affinity of RhlR for target DNA sequences. PqsE catalytic activity is dispensable for interaction with RhlR. Thus, the virulence function of PqsE can be decoupled from its catalytic function. Here, we present an immunoprecipitation-mass spectrometry method employing enhanced green fluorescent protein-PqsE fusions to define the protein interactomes of wild-type PqsE and the catalytically inactive PqsE(D73A) variant in P. aeruginosa and their dependence on RhlR. Several proteins were identified to have specific interactions with wild-type PqsE while not forming associations with PqsE(D73A). In the Δ*rhlR* strain, an increased number of specific PqsE interactors were identified, including the partner autoinducer synthase for RhlR, called RhlI. Collectively, these results suggest that specific protein-protein interactions depend on PqsE catalytic activity and that RhlR may prevent proteins from interacting with PqsE, possibly due to competition between RhlR and other proteins for PqsE binding. Our results provide a foundation for the identification of the *in vivo* PqsE catalytic function and, potentially, new proteins involved in P. aeruginosa quorum sensing.

## OBSERVATION

The opportunistic human pathogen Pseudomonas aeruginosa is responsible for causing highly antibiotic-resistant, virtually untreatable nosocomial infections (1, 2). P. aeruginosa pathogenic traits such as virulence factor production and biofilm formation are under the control of the bacterial cell-to-cell communication process called quorum sensing (QS) (3). QS relies on the production, release, and group-wide detection of signal molecules called autoinducers. The QS network in P. aeruginosa is composed of multiple interconnecting branches, including two acyl homoserine lactone autoinducer synthase/receptor transcription factor pairs, LasI/LasR and RhlI/RhlR (4). The RhlI/RhlR pair, responsible for producing and detecting, respectively, the C_4_-homoserine lactone (C_4_-HSL) autoinducer, controls cell density-dependent gene expression (5, 6). Curiously, given that RhlR and RhlI function in a receptor-ligand partnership, pathogenic phenotypes resulting from deletion of *rhlR* differ from those following deletion of *rhlI*. For instance, a Δ*rhlI* mutant can mount an infection in a murine host, whereas a Δ*rhlR* mutant is avirulent and does not establish infection (7). Deletion of the gene encoding the enzyme PqsE, which acts in a different P. aeruginosa QS pathway, results in the identical loss of pathogenic phenotypes as occurs following deletion of *rhlR* (8). Consistent with this result, we recently showed that PqsE and RhlR make a protein-protein interaction that enhances the affinity of RhlR for target DNA sequences (9, 10). Moreover, the PqsE-RhlR interaction is essential for RhlR-controlled QS phenotypes. Furthermore, the PqsE-RhlR interaction does not depend on PqsE catalytic activity, as the catalytically inactive PqsE(D73A) variant is capable of interacting with RhlR *in vitro* and driving virulence phenotypes in P. aeruginosa (9).

The *in vivo* role of PqsE catalytic function remains unknown. PqsE is encoded by the fifth gene in the *pqsABCDE* operon (11). PqsA to PqsE, together with PqsH (encoded separately in the genome), are responsible for synthesis of the Pseudomonas quinolone signal (PQS), a QS autoinducer (11, 12). However, a Δ*pqsE* mutant produces wild-type (WT) levels of PQS, and alternative thioesterases have been shown to fulfill the reported biosynthetic function of PqsE in this pathway (the conversion of 2-aminobenzoyl acetyl-coenzyme A [CoA] to 2-aminobenzoyl acetate) (13). Thus, beyond PqsE catalytic function being unnecessary for interaction with RhlR, it is also dispensable for production of PQS, highlighting the possibility that there exist yet unknown roles for PqsE-driven catalysis.

In this work, we explore the question of whether the PqsE-RhlR interaction occurs *in vivo* and whether PqsE participates in additional protein-protein interactions. To probe these possibilities, we determine the PqsE interactome in P. aeruginosa PA14. Another goal of this study is to gain insight into possible biosynthetic pathways requiring PqsE catalytic function. Here, we develop an immunoaffinity-mass spectrometry (IP-MS) strategy using PqsE tagged with monomeric enhanced green fluorescent protein (eGFP) as the bait protein. We employ both WT PqsE [designated PqsE(WT)] and a catalytically dead version harboring the D73A mutation [designated PqsE(D73A)] to distinguish PqsE interactions that require intact catalytic function from those that do not. The PqsE(WT) and PqsE(D73A) interactomes are identified in P. aeruginosa PA14 and in a Δ*rhlR* mutant strain. The results reveal a set of PqsE-protein interactions that depend on intact PqsE catalytic function. Furthermore, the Δ*rhlR* strain analyses show that the PqsE-RhlR interaction may prevent PqsE from interacting with a diverse set of proteins, including RhlI. These findings provide a platform to begin to address the yet unknown role (or roles) PqsE catalysis plays *in vivo* and to identify new proteins and mechanisms involved in the P. aeruginosa QS network.

### Designing the PqsE IP-MS workflow.

The PqsE-RhlR interaction is essential for P. aeruginosa to produce the toxin pyocyanin (9, 10). Thus, we monitored pyocyanin production to guide our design of a functional affinity-tagged PqsE fusion as the bait protein for IP-MS experiments to define the PqsE interactome. Both N-terminally (eGFP-PqsE) and C-terminally (PqsE-eGFP) tagged constructs were engineered into the pUCP18 vector and expressed in *ΔpqsE* P. aeruginosa PA14. Pyocyanin production was measured and compared to that from a strain carrying untagged PqsE on the same plasmid (see Fig. S1a in the supplemental material). The C-terminally tagged PqsE-eGFP construct drove significantly reduced pyocyanin production (22%, compared to the untagged construct), whereas the N-terminally tagged eGFP-PqsE construct showed only a small reduction in pyocyanin production (83%, compared to the untagged construct). This result is consistent with the finding that substitution of residues near the C terminus of PqsE (R243A/R246A/R247A) abolishes interaction with RhlR (10). Therefore, we used eGFP-PqsE constructs as bait in our IP-MS experiments. The eGFP tag in each construct was also confirmed to be folded and functional as judged by fluorescence output (see Fig. S1b). To control for interactions involving the eGFP tag, eGFP alone was also cloned into pUCP18 and identically assayed by IP-MS.

10.1128/mbio.01559-22.1 FIG S1 Functional testing of PqsE fusion constructs. (a) Pyocyanin production by strains harboring the pUCP18 vector expressing different *pqsE* genes fused to *eGFP*. Pyocyanin levels were determined by measuring the OD_695_ of cell-free culture fluids and dividing that value by the OD_600_ of the cell pellet that had been resuspended in PBS. The OD_695_/OD_600_ ratio of the strain carrying untagged PqsE was set to 100%. (b) Fluorescence from eGFP measured for cells from cell pellets that had been resuspended in PBS and normalized by the OD_600_. The minus sign represents the empty pUCP18 vector control. All experiments were performed in biological triplicate. Download FIG S1, PDF file, 0.03 MB. Copyright © 2022 Taylor et al. 2022 Taylor et al. https://creativecommons.org/licenses/by/4.0/ This content is distributed under the terms of the Creative Commons Attribution 4.0 International license.

Samples for IP-MS analyses were prepared using a strategy that enabled detection of weak and strong PqsE interactors. First, cryogenic grinding was employed for cell lysis, followed by rapid Polytron homogenization in a gentle lysis buffer optimized for preservation of protein complexes, taking into account considerations described previously (14, 15). Second, the protocol did not require chemical cross-linking. Third, the method relied on monomeric GFP (i.e., harboring the A206K substitution) as the affinity tag, eliminating formation of higher-order complexes that would be induced by multimerization of GFP. In analyses of the data from each IP-MS experiment, proteins were considered specific PqsE interactors if they were enriched by at least 2-fold compared to their abundance in the eGFP-alone sample, among other cutoff criteria that are described below. We know that PqsE interacts with RhlR; therefore, we performed this set of experiments in both WT and Δ*rhlR* P. aeruginosa strains to examine whether the presence/absence of RhlR influenced which specific interactions occur with PqsE. All strains used in this study are described in Table S1 in the supplemental material.

10.1128/mbio.01559-22.4 TABLE S1 Strains used in this study. Download Table S1, XLSX file, 0.01 MB. Copyright © 2022 Taylor et al. 2022 Taylor et al. https://creativecommons.org/licenses/by/4.0/ This content is distributed under the terms of the Creative Commons Attribution 4.0 International license.

### PqsE protein interactions that depend on catalytic function.

**FIG 1:**
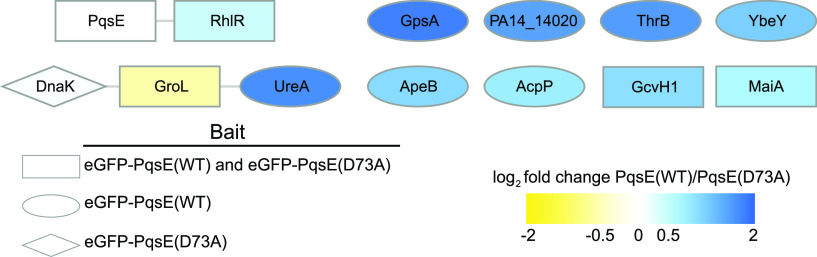
Specificity-filtered interactions with either eGFP-PqsE(WT) or eGFP-PqsE(D73A) in WT P. aeruginosa PA14. Shapes indicate whether the interaction passed specificity filtering for both bait proteins (rectangles), only eGFP-PqsE(WT) (ovals), or only eGFP-PqsE(D73A) (diamonds). Gray lines represent known or predicted interactions from the STRING database. Nodes are colored by abundance-based enrichment in the eGFP-PqsE(WT) (blue) or eGFP-PqsE(D73A) (yellow) IP experiments.

In WT P. aeruginosa PA14, 11 proteins were identified as enriched in the eGFP-PqsE(WT) IP-MS experiment, compared to that with eGFP alone (Fig. 1; also see Table S2). This result represents 0.2% of the annotated P. aeruginosa proteome and therefore indicates highly specific interactions. Indeed, RhlR was identified as an interacting partner for PqsE. RhlR was also identified in the eGFP-PqsE(D73A) IP-MS experiment, confirming that this interaction is independent of the PqsE catalytic function. In contrast, 7 of the 11 proteins identified in the eGFP-PqsE(WT) experiment did not pass specificity filtering in the eGFP-PqsE(D73A) IP-MS analysis (Fig. 1, ovals), suggesting that their interactions with PqsE depend on PqsE catalytic function. These 7 proteins are UreA, ThrB, GpsA, AcpP, ApeB, PA14_14020, and YbeY. Of these potential interacting partners, the acyl carrier protein AcpP is of particular interest because it participates in the synthesis of acyl homoserine lactone QS autoinducers (16). More generally, all of the proteins whose interactions with PqsE depended on its possessing intact catalytic function are enzymes, suggesting that their interactions with PqsE could potentially accomplish a biosynthetic function. In addition to RhlR, the proteins that interacted with both PqsE(WT) and the catalytically dead PqsE(D73A) protein were GroL, GcvH1, and MaiA. The molecular chaperone DnaK was observed as a specific interactor with PqsE(D73A) but not with PqsE(WT). Because GroL and DnaK are both chaperones, highly abundant, and promiscuous interactors, their interactions with PqsE are to be considered cautiously. It is also noteworthy that chaperones were more abundant in the IP experiments with eGFP-PqsE(D73A) than in those with eGFP-PqsE(WT), which could indicate that PqsE(D73A) is less stable than PqsE(WT). However, we showed previously that the cloned untagged proteins are produced with similar abundances in both P. aeruginosa and Escherichia coli; moreover, the two purified proteins have nearly equal melting temperatures (9), suggesting similar stabilities.

10.1128/mbio.01559-22.5 TABLE S2 All P. aeruginosa proteins identified in the WT GFP-PqsE IPs. Download Table S2, XLSX file, 0.2 MB. Copyright © 2022 Taylor et al. 2022 Taylor et al. https://creativecommons.org/licenses/by/4.0/ This content is distributed under the terms of the Creative Commons Attribution 4.0 International license.

### RhlR inhibits other proteins from interacting with PqsE.

**FIG 2:**
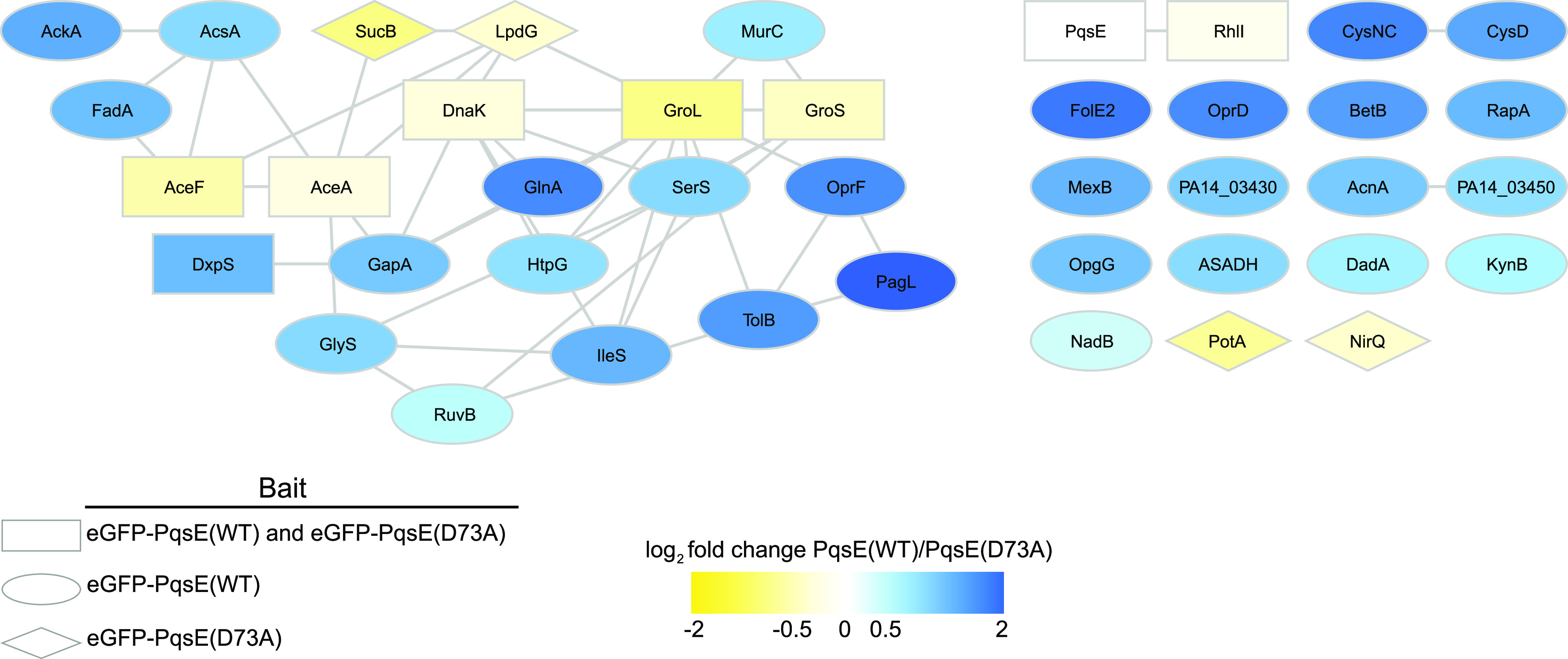
Specificity-filtered interactions with eGFP-PqsE(WT) or eGFP-PqsE(D73A) in the P. aeruginosa PA14 Δ*rhlR* strain. Shapes indicate whether the interaction passed specificity filtering for both bait proteins (rectangles), only eGFP-PqsE(WT) (ovals), or only eGFP-PqsE(D73A) (diamonds). Gray lines represent known or predicted interactions from the STRING database. Nodes are colored by abundance-based enrichment in the eGFP-PqsE(WT) (blue) or eGFP-PqsE(D73A) (yellow) IP experiments.

When the IP-MS experiment described above was conducted with eGFP-PqsE(WT) in *ΔrhlR* P. aeruginosa PA14, surprisingly, the number of PqsE-specific interacting proteins increased to 36 (Fig. 2, rectangles and ovals; also see Table S3), 13 of which were at least 2-fold more abundant in the Δ*rhlR* strain IP than in the WT P. aeruginosa PA14 IP (Table 1; also see Fig. S2a, blue nodes). With the exceptions of GroL and DnaK, none of the proteins that passed specificity filtering in WT P. aeruginosa PA14 were identified as specific PqsE interactors in the Δ*rhlR* strain. We note particularly that, in *ΔrhlR* P. aeruginosa PA14, both eGFP-PqsE(WT) and eGFP-PqsE(D73A) interacted with the C_4_-HSL synthase RhlI (Fig. 2 and Tables 1 and 2; also see Fig. S2a and b). This finding suggests that the potential PqsE-RhlI interaction is independent of PqsE catalytic function. In contrast to RhlI, several of the eGFP-PqsE(WT) interactors identified in the Δ*rhlR* strain did not pass filtering in the Δ*rhlR* strain with eGFP-PqsE(D73A) as the bait (Fig. 2, ovals), suggesting that these interactions depend on PqsE catalytic function.

**TABLE 1:** Log_2_ fold change comparison between PqsE(WT) IPs in WT and Δ*rhlR* P. aeruginosa strains

Protein accession no.	Gene	Displayed gene name	WT/Δ*rhlR* strain log_2_ abundance ratio
P54292	*rhlR*	*rhlR*	9.965784285
Q02SF0	*ybeY*	*ybeY*	2.956800118
Q02LM8	*kynB*	*kynB*	1.616357697
Q02DL4	*thrB*	*thrB*	1.365692537
Q02FF4	*ureA*	*ureA*	1.264836648
Q9I3F5	*acnA*	*acnA*	−1.002888279
Q02V73	*glyS*	*glyS*	−1.008682243
Q59638	*aceF*	*aceF*	−1.067938829
B7UZY2	*cysD*	*cysD*	−1.092340172
Q02H29	*murC*	*murC*	−1.224317298
P27726	*gap*	*gapA*	−1.354759487
Q51363	*nadB*	*nadB*	−1.354759487
Q9I6M4	*davT*	PA14_03450	−1.411195433
B7UYA0	*ackA*	*ackA*	−1.486004021
P30718	*groL*	*groL*	−1.708396442
Q02H54	*groS*	*groS*	−1.76611194
P20581	*pqsE*	*pqsE*	−2.005782353
P54291	*rhlI*	*rhlI*	−9.965784285

**TABLE 2:** Log_2_ fold change comparison between PqsE(D73A) IPs in WT and Δ*rhlR* P. aeruginosa strains

Protein accession no.	Gene	Displayed gene name	WT/Δ*rhlR* strain log_2_ abundance ratio
P54292	*rhlR*	*rhlR*	3.863343811
P57109	*maiA*	*maiA*	1.052415894
P54291	*rhlI*	*rhlI*	−0.962969269
P20581	*pqsE*	*pqsE*	−1.373327247
Q59637	*aceE*	*aceA*	−1.392137097
P30718	*groL*	*groL*	−1.415037499
Q02H54	*groS*	*groS*	−1.60823228
Q9I3D2	*sucB*	*sucB*	−1.694321257
Q59638	*aceF*	*aceF*	−1.805912948
Q51481	*nirQ*	*nirQ*	−5.158429363

10.1128/mbio.01559-22.2 FIG S2 Specificity-filtered interactions with eGFP-PqsE(WT) (a) and eGFP-PqsE(D73A) (b) in either WT or Δ*rhlR* P. aeruginosa. Shapes indicate whether the interaction was specific in both the WT and Δ*rhlR* strains (diamonds), in only the WT strain (ovals), or in only the Δ*rhlR* strain (rounded rectangles). Gray lines represent known or predicted interactions from the STRING database. Nodes are colored by abundance-based enrichment in the WT strain (red) or Δ*rhlR* strain (blue) PqsE IP experiments. Download FIG S2, PDF file, 0.1 MB. Copyright © 2022 Taylor et al. 2022 Taylor et al. https://creativecommons.org/licenses/by/4.0/ This content is distributed under the terms of the Creative Commons Attribution 4.0 International license.

10.1128/mbio.01559-22.6 TABLE S3 All P. aeruginosa proteins identified in the Δ*rhlR* GFP-PqsE IPs. Download Table S3, XLSX file, 0.2 MB. Copyright © 2022 Taylor et al. 2022 Taylor et al. https://creativecommons.org/licenses/by/4.0/ This content is distributed under the terms of the Creative Commons Attribution 4.0 International license.

To investigate whether the increased number of PqsE interactors identified in the Δ*rhlR* strain, compared to the WT strain, stemmed from increases in their protein abundances in the Δ*rhlR* strain, we conducted whole-proteome analyses. Only NirQ, GapA, and AcpP were more abundant in the Δ*rhlR* strain lysate than in the WT lysate (see Tables S4 and S5 and Fig. S3). Thus, we conclude that it is the absence of RhlR, and not the upregulation of genes that are normally repressed by RhlR, that leads to the increase in specific interactions of proteins with PqsE. One note about AcpP is that it specifically interacted with PqsE only in WT P. aeruginosa. Thus, the finding that its abundance increased in the Δ*rhlR* strain suggests that RhlR could be required for formation of a PqsE-RhlR-AcpP complex. This possibility and the possibility that RhlR is required for complex formation with other PqsE-interacting partners are currently being investigated.

10.1128/mbio.01559-22.3 FIG S3 Waterfall plots showing log_2_ fold changes in proteins in WT and Δ*rhlR* strains for GFP (a), PqsE(WT) (b), and PqsE(D73A) (c). All proteins detected in the whole-proteome analyses are shown in gray, and proteins identified as putative PqsE interactors in the IP analyses are shown in blue. An absolute log_2_ fold change value of 1 was used as the criterion for significant fold changes in protein abundances, as indicated by the dashed lines. Representative proteins identified as altered in abundance between the two strains in the IP analyses are indicated. Download FIG S3, PDF file, 0.7 MB. Copyright © 2022 Taylor et al. 2022 Taylor et al. https://creativecommons.org/licenses/by/4.0/ This content is distributed under the terms of the Creative Commons Attribution 4.0 International license.

10.1128/mbio.01559-22.7 TABLE S4 Whole-proteome analyses of all strains in this study. Download Table S4, XLSX file, 0.8 MB. Copyright © 2022 Taylor et al. 2022 Taylor et al. https://creativecommons.org/licenses/by/4.0/ This content is distributed under the terms of the Creative Commons Attribution 4.0 International license.

10.1128/mbio.01559-22.8 TABLE S5 Abundance of differentially interacting proteins in WT PA14 versus Δ*rhlR* PA14, defined as the combination of proteins illustrated in Fig. 1 and 2. Download Table S5, XLSX file, 0.01 MB. Copyright © 2022 Taylor et al. 2022 Taylor et al. https://creativecommons.org/licenses/by/4.0/ This content is distributed under the terms of the Creative Commons Attribution 4.0 International license.

### Discussion.

PqsE plays an essential role in driving pathogenic behaviors in P. aeruginosa PA14, highlighting the importance of defining its *in vivo* function. We know that to activate virulence, PqsE makes a protein-protein interaction with the QS regulator RhlR and this interaction does not rely on PqsE catalytic activity. The PqsE *in vivo* catalytic function, as well as any additional protein-protein interactions, remains unknown. Here, we engineered a functional (i.e., capable of driving pyocyanin production) (see Fig. S1a) PqsE protein with eGFP fused to the N terminus for use in the *in vivo* IP-MS experiments. Future experiments could employ PqsE constructs with eGFP fused to the C terminus to identify interactions involving the N terminus of PqsE. Through our IP-MS analyses, we identified additional PqsE interactions that occur in P. aeruginosa PA14, and we can distinguish those that require intact PqsE catalytic function from those that do not. Proteins that interact with PqsE(WT) but not the catalytically dead PqsE(D73A) variant could be indicative of biosynthetic pathways in which PqsE participates. Notably, the AcpP acyl carrier protein was identified, as was the RhlI C_4_-HSL synthase, in the Δ*rhlR* data set. These findings hint that the annotated thioesterase function of PqsE could be important for editing acyl chain length during the synthesis of acyl homoserine lactone autoinducers such as C_4_-HSL. We are currently pursuing metabolomic analyses to identify small-molecule substrates and products of PqsE.

The interaction between PqsE and RhlR was previously established through mutagenesis of PqsE combined with *in vitro* pulldown assays using recombinant proteins produced in E. coli (9, 10). Here, we validate that the PqsE-RhlR interaction occurs in P. aeruginosa PA14 *in vivo* and that it is independent of PqsE catalytic function. Surprisingly, we also show that PqsE interacts with many more proteins in the Δ*rhlR* strain than in WT P. aeruginosa PA14. These putative PqsE interactors harbor a wide diversity of functions. This result suggests that interaction between PqsE and RhlR is primary, and it blocks interactions that PqsE can undertake with other proteins, including RhlI. The phenotypes of Δ*rhlR* and Δ*rhlI* P. aeruginosa mutants differ significantly (7). Our observation that PqsE-specific protein interactions, including with RhlI, occur in the Δ*rhlR* mutant but cannot be detected in WT P. aeruginosa hints at a potential mechanism underlying these phenotypic differences. Perhaps RhlI and RhlR compete for interaction with PqsE, and their relative production levels under different conditions determine which complex forms. Going forward, it is of interest to perform analogous IP-MS experiments in a Δ*rhlI* P. aeruginosa mutant. Although the PqsE interactions reported here remain to be validated, the results of this study provide the starting point for exploring the *in vivo* functions of this vital component of the P. aeruginosa QS and pathogenesis networks.

### Growth conditions and sample preparation.

We use the designation PA14 to signify P. aeruginosa UCBPP-PA14. All strains used in this study are listed in Table S1 in the supplemental material. The following six strains were grown as overnight cultures in LB supplemented with carbenicillin (400 μg/mL): WT PA14 plus pUCP18_eGFP, WT PA14 plus pUCP18_eGFP-PqsE(WT), WT PA14 plus pUCP18_eGFP-PqsE(D73A), Δ*rhlR* PA14 plus pUCP18_eGFP, Δ*rhlR* PA14 plus pUCP18_eGFP-PqsE(WT), and Δ*rhlR* PA14 plus pUCP18_eGFP-PqsE(D73A). The overnight cultures were back-diluted 1:100 into 25 mL fresh LB with carbenicillin and grown at 37°C with shaking (200 rpm) until they reached an optical density at 600 nm (OD_600_) of 1.5. Cells were pelleted by centrifugation at 4,000 rpm for 15 min, washed three times with 5 mL phosphate-buffered saline (PBS), resuspended in 200 μL freezing buffer (20 mM HEPES, 1.2% polyvinylpyrrolidone [wt/vol] [pH 7.4]), and flash-frozen by slow pipetting of droplets into liquid nitrogen. The frozen droplets were stored at −80°C until cryogenic grinding. Cryogenic grinding was performed in a Retsch CryoMill with nine cycles at a frequency of 30 Hz, lasting 1.5 min per cycle. After grinding, the frozen cell powders were transferred into prechilled LoBind tubes (Amuza, Inc., USA) and stored at −80°C until lysis and IP were performed. All samples used for IP analysis were collected in biological triplicate. All samples used for whole-proteome analysis were collected in biological duplicate.

### Lysis and IP.

The frozen cell powders were resuspended in prechilled lysis buffer (20 mM HEPES [pH 7.4], 100 mM potassium acetate, 2 mM MgCl_2_, 0.1% Tween 20 [vol/vol], 1 μM ZnCl_2_, 1 μM CaCl_2_, 1% Triton X-100 [vol/vol], 200 mM NaCl, 0.5 mM phenylmethylsulfonyl fluoride [PMSF], 1:2,500 Pierce universal nuclease, with a protease inhibitor cocktail [Roche] [one tablet/10 mL buffer]). Resuspension was carried out by inversion and gentle vortex-mixing, followed by rotation for 30 min at 4°C until the samples were completely solubilized. Lysates were subsequently subjected to Polytron homogenization by pulsing twice for 15 s at a speed of 22,500 rpm. Samples were incubated on ice for 10 s between pulses. Lysates were cleared by centrifugation at 10,000 × *g* at 4°C for 10 min. Protein concentrations were determined by bicinchoninic acid (BCA) analysis, and 500 μg of protein from each sample was incubated with 35 μL prewashed GFP-Trap magnetic beads (Chromotek, Inc.) for 1 h at 4°C with rotation (final lysate concentration of 1 mg/mL). The beads were separated from the total sample using a magnet, and supernatant was removed by aspiration. The beads were washed three times with 500 μL wash buffer (lysis buffer lacking PMSF, nuclease, and protease inhibitors). A final wash with cold PBS was performed, and the beads were transferred to a new tube. Proteins were eluted in 50 μL 1× TES buffer (2% SDS, 0.5 mM EDTA, 53 mM Tris-HCl, 70 mM Tris base) by incubation at 70°C for 10 min and vortex-mixing for 20 s. The eluate was transferred to a new Lo-Bind tube. For whole-proteome samples, the same lysis buffer was employed, and resuspension was performed as described above. The cells were further lysed by sonication and then cleared by centrifugation at 10,000 × *g* at 4°C for 10 min. Methanol-chloroform extraction was performed on the clarified lysates. Briefly, methanol, chloroform, and high-performance liquid chromatography (HPLC)-grade water were sequentially added to the lysates at a ratio of 4:1:3 (relative to sample volume). The mixtures were sonicated and subjected to centrifugation at 15,000 × *g* at room temperature. Liquids in the tubes were aspirated, and the protein disks were washed once with 3 volumes of ice-cold MS-grade methanol and a second time with 5 volumes of ice-cold MS-grade methanol. The pellets were air dried and resuspended in 50 μL 1× TES buffer (2% SDS, 0.5 mM EDTA, 53 mM Tris-HCl, 70 mM Tris base). Protein concentrations were determined by BCA analysis, and 50 μg of protein from each sample was reduced and alkylated with 25 mM Tris(2-carboxyethyl)phosphine (TCEP) (Thermo Fisher Scientific) and 50 mM chloroacetamide (CAM) (Thermo Fisher Scientific) by heating at 70°C for 20 min.

### Preparation of samples for MS analysis.

IP eluates were concentrated 2-fold in a SpeedVac concentrator. Proteins were reduced and alkylated with 25 mM TCEP (Thermo Fisher Scientific) and 50 mM CAM (Thermo Fisher Scientific) by heating at 70°C for 20 min. The resulting samples were digested using an S-Trap microcolumn (ProtiFi, LLC), as described previously (17). Briefly, samples were acidified to 1.2% phosphoric acid, diluted into S-Trap binding buffer (100 mM triethylammonium bicarbonate [TEAB] [pH 7.1] in 90% methanol), and bound to the S-Trap column by centrifugation at 4,000 × *g* for 30 s. The S-Trap-bound sample was washed using the same centrifugation procedure, as follows: two washes with S-Trap binding buffer, five washes with methanol-chloroform (4:1 [vol/vol]), and three washes with S-Trap binding buffer. Samples were next digested on the S-Trap column for 1 h at 47°C with 2.5 μg trypsin diluted in digestion buffer (25 mM TEAB). Trypsinized peptides were eluted through a three-part elution (40 μL 25 mM TEAB, 40 μL 0.2% formic acid [FA], and 70 μL 50% acetonitrile with 0.2% FA) by sequentially adding the elution buffers to the column, followed by centrifugation as described above after addition of each buffer. The eluted peptides were pooled in a liquid chromatography (LC)-MS autosampler vial (Thermo Fisher Scientific) and dried with a SpeedVac concentrator. Peptides were resuspended in 6 μL 1% FA-1% acetonitrile. Whole-proteome samples were digested using an S-Trap column as described above.

### MS and data acquisition.

IP samples were analyzed on a Q-Exactive HF mass spectrometer (Thermo Fisher Scientific) equipped with a Nanospray Flex ion source (Thermo Fisher Scientific). Peptides were separated on a 50-cm column (inner diameter, 360 μm; outer diameter, 75 μm; Thermo Fisher Scientific) packed in-house with ReproSil-Pur C_18_ resin (pore size, 120 Å; particle size, 1.9 μm; ESI Source Solutions). Peptides were separated over a 150-min gradient of 3% solvent B to 35% solvent B (solvent A, 0.1% FA; solvent B, 0.1% FA-97% acetonitrile) at a flow rate of 0.25 nL/min. MS1 scans were collected with the following parameters: resolution, 120,000; maximum injection time (MIT), 30 ms; automatic gain control (AGC), 3*e*6; scan range, *m/z* 350 to 1,800; data collection mode, profile. MS2 scans were collected with the following parameters: resolution, 30,000; MIT, 150 ms; AGC, 1*e*5; isolation window, *m/z* 1.6; loop count, 10, normalized collision energy (NCE), 28; fixed first mass, *m/z* 100.0; peptide match, preferred; data collection mode, centroid; dynamic exclusion, 45 s. Whole-cell lysate samples were analyzed on the same instrument and column type. Peptides were separated over a 110-min gradient of 3% solvent B to 30% solvent B at a flow rate of 0.25 μL/min. MS1 scans were collected with the following parameters: resolution, 120,000; MIT, 30 ms; AGC, 3*e*6; scan range, *m/z* 350 to 1,800; data collection mode, profile. MS2 scans were collected with the following parameters: resolution, 15,000; MIT, 25 ms; AGC, 1*e*5; isolation window, *m/z* 1.2; loop count, 20; NCE, 27; fixed first mass, *m/z* 150.0; peptide match, preferred; data collection mode, centroid; dynamic exclusion, 30 s.

### MS data analysis.

MS/MS spectra were analyzed in Proteome Discoverer v.2.4 (Thermo Fisher Scientific). SEQUEST HT was used to search spectra against a UniProt database containing P. aeruginosa protein sequences (downloaded October 2020) and common contaminants. Offline mass recalibration was performed via the Spectrum Files RC node, and the Minora Feature Detector node was used for label-free MS1 quantification. Fully tryptic peptides with a maximum of two missed cleavages, a precursor mass tolerance of 4 ppm, and a fragment mass tolerance of 0.02 Da were used in the search. Posttranslational modifications (PTMs) that were allowed included the static modification carbamidomethylation of cysteine and the following dynamic modifications: oxidation of methionine, deamidation of asparagine, loss of methionine plus acetylation of the N terminus of the protein, acetylation of lysine, and phosphorylation of serine, threonine, and tyrosine. Peptide spectrum match (PSM) validation was accomplished using the Percolator node, and PTM sites were assigned in the ptmRS node. PSMs were assembled into peptide and protein identifications with a false discovery rate of less than 1% at both the peptide and protein levels, with at least two unique peptides identified per protein. Regarding IP samples, precursor quantitation required identification in at least two of the three replicates. Samples were normalized in a retention time-dependent manner, imputation was performed using low-abundance resampling, protein abundances were calculated using summed abundances, and protein ratio calculations were performed using pairwise ratios. Regarding whole-proteome samples, quantified proteins were reported if two unique peptides were detected in both replicates of at least one sample group and had <75% coefficients of variance for all sample groups. Raw and adjusted *P* values were calculated by Proteome Discoverer using the *t* test (background) method, which contrasts individual protein ratios to the background of all quantified proteins. Differential proteins were assigned if the absolute value of the log_2_ protein ratio was higher than 1 and the adjusted *P* value was less than 0.05.

Data were exported to Microsoft Excel for further processing. For the IP data analyses, in order for a protein to be considered a putative PqsE interactor, the following requirements had to be met: (i) there must be at least a 2-fold enrichment of the protein in the sample of interest compared to the eGFP control, (ii) at least two peptides had to be quantified in the sample of interest [PqsE(WT) or PqsE(D73A)] in all replicates, and (iii) the grouped coefficient of variation (CV) had to be less than 75%. To compare PqsE(WT) and PqsE(D73A) interactions, bait normalization was performed by dividing the PqsE(WT)/PqsE(D73A) abundance ratio for each interacting protein by the bait abundance ratio for PqsE(WT)/PqsE(D73A). Fold changes of 1.5 or higher were considered significant. Bait normalization was performed only for interactions that passed specificity filtering, as described above. To compare interactions between the WT and Δ*rhlR* strains, bait normalization could not be performed; therefore, the WT/Δ*rhlR* abundance ratio was calculated, ratios for proteins identified exclusively in one sample were manually verified, and a stringent cutoff value for fold changes of 2 or higher was considered significant. Protein interaction networks were generated using STRING v.11 (18) and Cytoscape v.3.8.2 (19). Little experimental information exists concerning protein-protein interactions in P. aeruginosa. For this reason, to analyze our data, we used a broad STRING analysis that included predicted interactions from experiments, databases, coexpression, neighborhood, gene fusion, cooccurrence, and text mining.

### Data availability.

The MS proteomic data have been deposited at the ProteomeXchange Consortium via the PRIDE partner repository (20) with the data set identifier PXD035779.
