# Supplementary figures and images for: Protein Interaction Networks of Catalytically Active and Catalytically Inactive PqsE in Pseudomonas aeruginosa
Source: mBio. 2022 Sep 8;13(5):e01559-22. doi: 10.1128/mbio.01559-22 (PMC9600345; doi:10.1128/mbio.01559-22)

**a**

### Pyocyanin Production

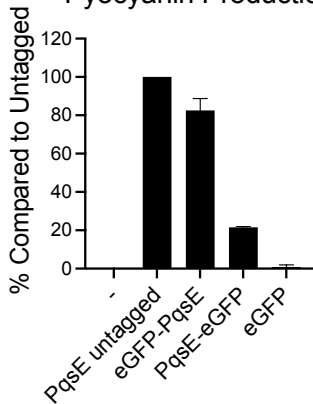**b**

### eGFP Fluorescence

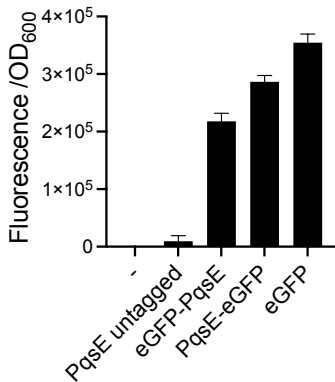

Supplement: FIG S1 [file mbio.01559-22-s0001.pdf]

a) GFP

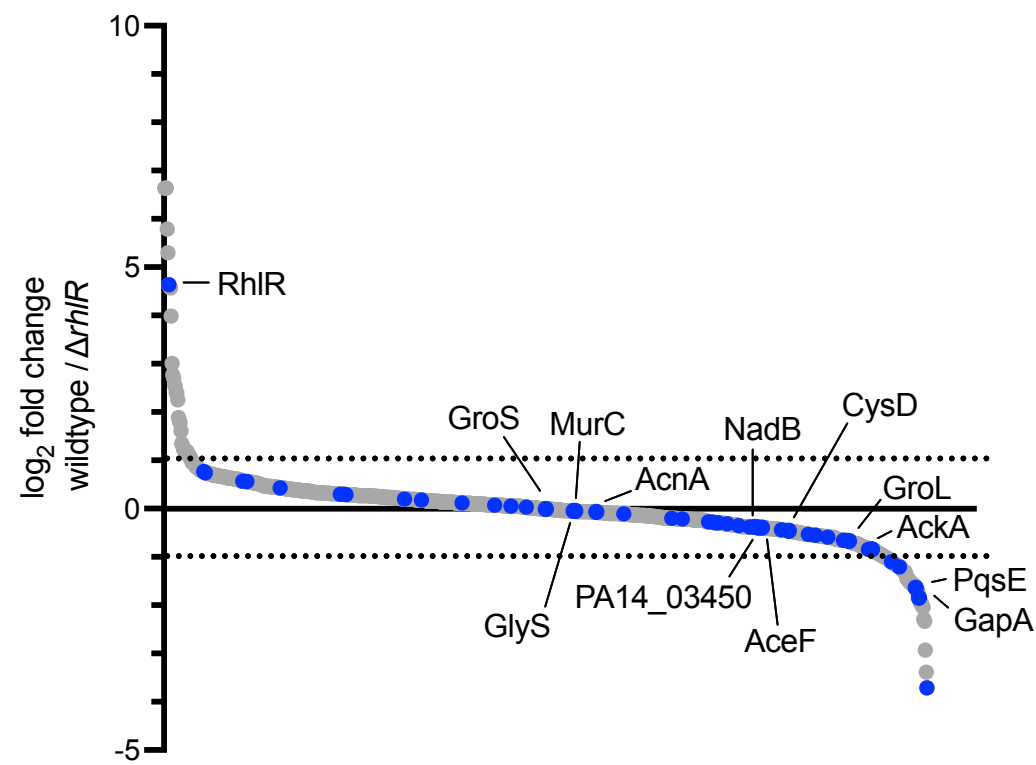

b) PqsE(WT)

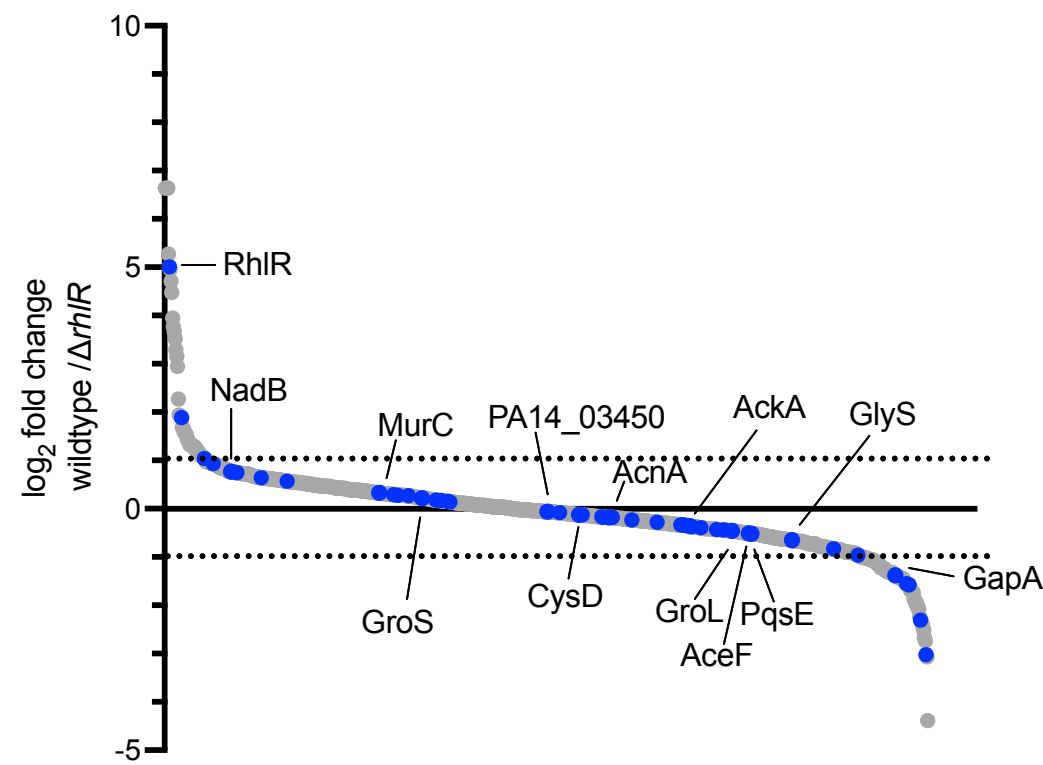

c) PqsE(D73A)

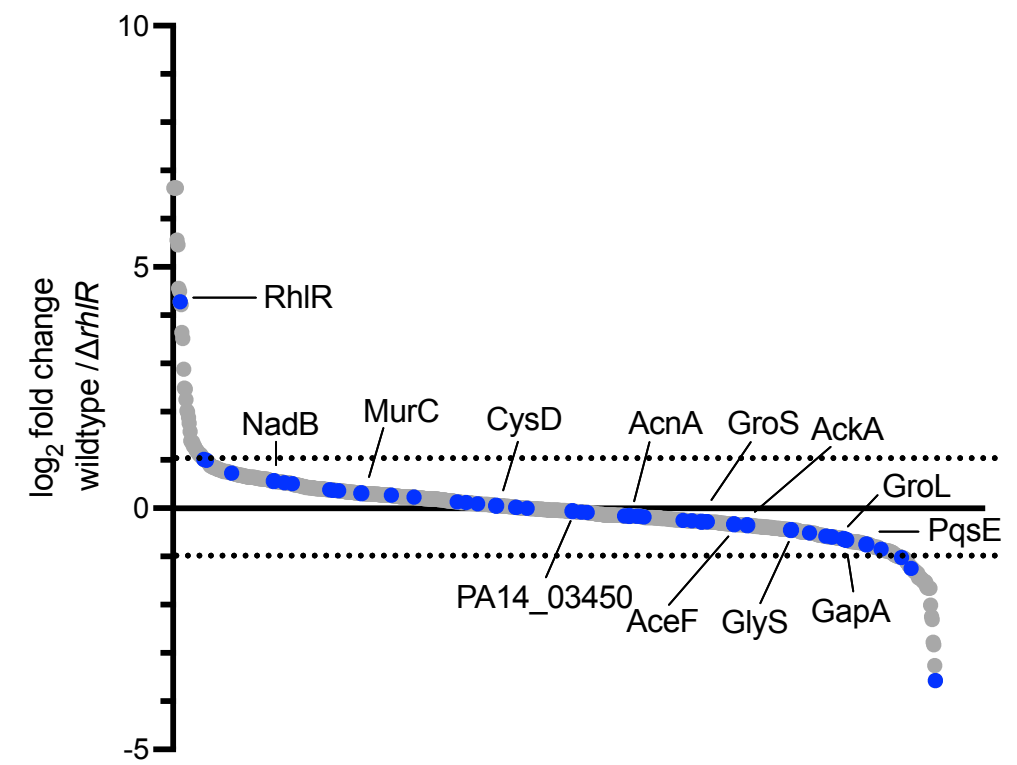

Supplement: FIG S3 [file mbio.01559-22-s0003.pdf]
