# Supplementary material for: Protein Interaction Networks of Catalytically Active and Catalytically Inactive PqsE in Pseudomonas aeruginosa
Source: mBio. 2022 Sep 8;13(5):e01559-22. doi: 10.1128/mbio.01559-22 (PMC9600345; doi:10.1128/mbio.01559-22)

a) eGFP-PqsE(WT) interactions in wildtype and Δ*rhlR* *P. aeruginosa*

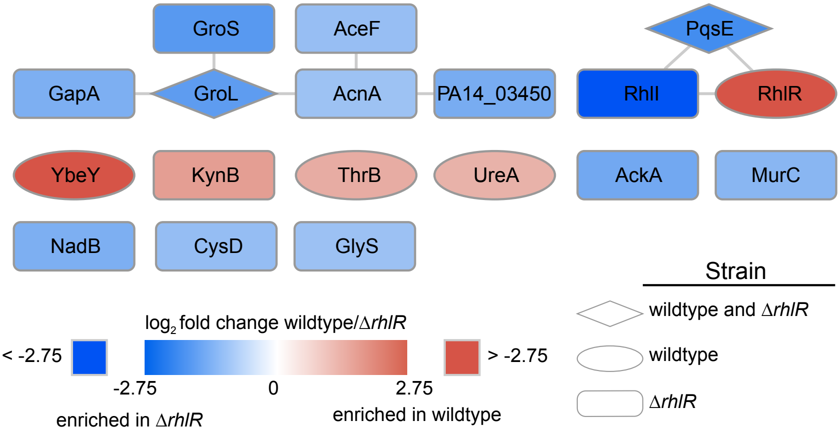

b) eGFP-PqsE(D73A) interactions in wildtype and Δ*rhlR* *P. aeruginosa*

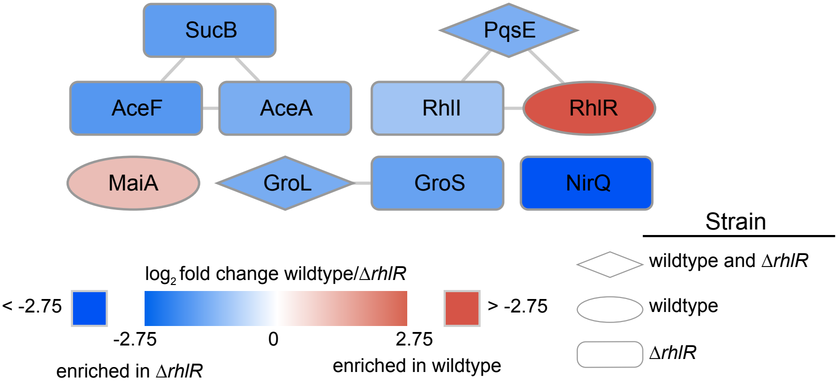

Supplement: FIG S2 [file mbio.01559-22-s0002.pdf]
